# Immunosenescence of Polymorphonuclear Neutrophils

**DOI:** 10.1100/tsw.2010.14

**Published:** 2010-01-21

**Authors:** Inga Wessels, Judith Jansen, Lothar Rink, Peter Uciechowski

**Affiliations:** Institute of Immunology, Medical Faculty RWTH Aachen University, Germany

**Keywords:** neutrophils, polymorphonuclear neutrophils, immunosenescence, inflammaging, aging, elderly, SENIEUR protocol, adhesion, polymorphonuclear leukocytes, phagocytosis, chemotaxis, degranulation, intracellular killing, inflammation, apoptosis, G-CSF, GM-CSF, fMLP, toll-like receptor, triggering receptor expressed on myeloid cells, signaling, membrane fluidity

## Abstract

All immune cells are affected by aging, contributing to the high susceptibility to infections and increased mortality observed in the elderly. The effect of aging on cells of the adaptive immune system is well documented. In contrast, knowledge concerning age-related defects of polymorphonuclear neutrophils (PMN) is limited. During the past decade, it has become evident that in addition to their traditional role as phagocytes, neutrophils are able to secrete a wide array of immunomodulating molecules. Their importance is underlined by the finding that genetic defects that lead to neutropenia increase susceptibility to infections. Whereas there is consistence about the constant circulating number of PMN throughout aging, the abilities of tissue infiltration, phagocytosis, and oxidative burst of PMN from aged donors are discussed controversially. Furthermore, there are numerous discrepancies between *in vivo* and *in vitro* results, as well as between results for murine and human PMN. Most of the reported functional changes can be explained by defective signaling pathways, but further research is required to get a detailed insight into the underlying molecular mechanisms. This could form the basis for drug development in order to prevent or treat age-related diseases, and thus to unburden the public health systems.

## INTRODUCTION

Certain tumors, autoimmune disorders, and infectious diseases contribute to increased mortality and morbidity in aged individuals. The higher susceptibility to and delayed recovery from severe infections, as well as the decreased response to vaccination, are consequences, in part, of the so-called "immunosenescence". Immunosenescence is defined as decreased cellular reactivity, and imbalance between inflammatory and anti-inflammatory networks, resulting in the low-grade, chronic, proinflammatory status also known as "inflammaging"[1]. The worldwide increase of the proportion of people older than 65 years has led to the rising costs of age-related diseases experienced by public health services and enhanced the interest in research on immunosenescence during the past decade. A better understanding of immunosenescence could help to limit the development and progression of age-related diseases, such as Alzheimer's disease, arteriosclerotic diseases, or cardiovascular diseases. The complexity of the network of cells and soluble factors, strictly balanced and regulated to create a successful immune response, is a great challenge for scientists and, at the same time, a problem for physicians.

To separate age-related changes from alterations due to nutrition, lifestyle, medications, or underlying diseases, elderly donors for the study of immunosenescence need to be recruited according to the SENIEUR protocol, which discriminates between healthy and frail elderly people[2]. While the effects of aging on T and B lymphocytes are extensively studied, polymorphonuclear neutrophils (PMN) are often neglected by scientists, even though PMN play an important role in repairing and destroying tissues, through their own action and also via their influence on the adaptive immune response. They give instructions to promote as well as to restrain inflammation, and offer potential opportunities for pharmacological interference in order to treat and prevent age-related immune deficiencies[3]. This review highlights the age-related changes of the immune system, focusing on new insights into neutrophil immunity.

## THE AGED IMMUNE SYSTEM

To create an efficient immune response, coordination of a complex network of cells and soluble factors is essential. These factors and cells can be grouped into the innate and adaptive part of the immune system. The cellular basis of both parts is formed by hematopoietic stem cells (HSC). These HSC and/or their microenvironment usually balance the initial differentiation steps according to differentiated cell output requirements[4]. The number of human $CD34^+$ hematopoietic progenitors in the elderly is reduced[5,6]. The first analyses of molecular changes in HSC during aging in mice showed an up-regulation of myeloid-specific genes and myeloid leukemia genes, a reduced expression of lymphoid genes, changes in genes linked to epigenetic regulation, as well as changes in cell adhesion genes[7]. These data support the finding that HSC from aged donors do not efficiently generate T- and B-lymphoid progenitors (common lymphoid progenitors, CLP), whereas they are superior in supporting the myeloid cell lineage[8]. Both the generated CLP and the myeloid type of progenitor cells show reduced proliferation potential[9,10]. In summary, the alterations of aged stem cells seem to correlate with immunosenescence.

Immunosenescence of the lymphoid lineage is not the focus of this review and is extensively summarized elsewhere[11,12,13,14,15,16].

Monocytes, as a part of the innate immune system, originate from the same myeloid stem cell progenitors as granulocytes and differentiate into macrophages in a variety of tissues. Interestingly, they share some functions with PMN and these functions are altered in both cell types by aging. Early studies concerning the effects of aging on monocytes often show conflicting results. Some show an increase of lipopolysaccharide (LPS)-induced cytokine production[17,18,19,20], whereas others claim the contrary[21,22]. The loss of several other monocytic functions has been shown more consistently. Those include the loss of toll-like receptor (TLR)1/2–induced interleukin (IL)-6 and tumor necrosis factor (TNF)-$\alpha$ production, partly explained by the diminished surface expression of TLR1 and TLR4[22], and the decrease of TLR-induced CD80 up-regulation. Phagocytosis is also impaired[22]. A recent study provides evidence for age-associated defects in TLR function in macrophages. More precisely, macrophages from elderly persons have a defective signaling pathway, resulting in an inability to down-regulate TLR3 during West Nile virus infections, which could explain the diminished success in fighting viral infections[23].

Finally, age-related changes of dendritic cells (DC), the major antigen-presenting innate immune cells, have also been detected. DC can be subdivided into plasmacytoid DC (pDC) and myeloid DC (mDC), but both belong to the myeloid lineage[24]. There are no changes in their number, TLR expression, or TLR function, but migration and chemotaxis are impaired in DC from aged donors. Phago- and pinocytosis are also disturbed[25], finally leading to a decreased ability of presenting foreign, but also self, antigens, which is necessary for maintenance of peripheral tolerance[26,27,28]. Taken together, this provides an explanation for the disrupted interaction with B cells[29,30]. While some groups report similar T-cell stimulation capacities of DC derived from young and aged donors[31,32], others report decreased priming in the lymph nodes of aged subjects[33,34,35,36]. DC of aged volunteers contribute to the constant proinflammatory status of the elderly by increased IL-6 and TNF-α production[37].

## NEUTROPHILS

PMN are key players of the innate immune system and belong to the first cells present at the site of infection. Their bactericidal and antifungal armory includes the generation of reactive oxygen and nitrogen species to kill phagocytosed pathogens, as well as the release of a range of proteases and antimicrobial peptides to attack extracellular pathogens[38,39]. With a half-life circulation time in blood of only 8–12 h[38,40], they are short-lived cells, but because of the continuous production of $1–2 \times 10^{11}$ PMN per day, they account for 60–70% of blood leukocytes[41]. However, they are often neglected in comparison to monocytes because it was long believed that their only functions were phagocytosis and oxidative burst. Current studies uncovered the secretion of an impressive array of mediators by neutrophils, even though they are end-differentiated cells with few endoplasmatic reticulum[42].

PMN are activated by compounds that bind to receptors that recognize pathogen-associated molecular patterns (PAMPs), such as formyl-methionyl-leucocyl-phenylalanin (fMLP), endotoxin, and other TLR ligands. Alternatively, they can also be activated by cytokines such as granulocyte-monocyte-colony stimulating factor (GM-CSF), IL-15, IL-18, or ligands of the recently discovered triggering receptor expressed on myeloid cells (TREM)-1[43,45]. The appropriate initiation, but also resolution, of their inflammatory response is crucial for the clearance of infections and the prevention of nonspecific tissue damage, which otherwise lead to chronic inflammatory disease and frailty. Neutrophil production is up-regulated during infection, leading to neutrophilia[40].

The life span of PMN is extended in response to granulocyte-colony stimulating factor (G-CSF), GM-CSF, complement factors, platelet activating factor (PAF), and proinflammatory cytokines (TNFα, IL-6, IL-1β)[38,46,47]. A hypoxic environment can also function as a survival factor[48]. The finding that neutropenia due to genetic defects or chemotherapy leads to increased susceptibility to bacterial and fungal infections[43,46] underlines the importance of effective PMN for the immune system.

## NEUTROPHILS DURING AGING

Several studies propose that the function of neutrophils might be compromised in the elderly. This is indicated not only by the increase of morbidity and mortality due to bacterial infections during aging[49], but also by a mass of clinical data showing that age is an independent risk factor for the development of chronic inflammatory diseases.

A lot of these diseases can be explained by functional disturbances of PMN. Recurring infections of the skin and respiratory tract due to the compromised bactericidal activity and chemotaxis of the PMN in the elderly should also be mentioned[46]. Table 1 presents an overview of the effect of aging on different functions of PMN. It also offers some possible underlying molecular mechanisms that could explain functional changes of PMN derived from aged donors.

### Proliferation and Apoptosis

The proliferative response of PMN precursors from aged donors to G-CSF is disrupted[39,50]. However, the intact responses to IL-3 and GM-CSF[51,52] are sufficient to ensure an adequate neutrophilia during infection. Total numbers of neutrophils remain constant throughout aging[51,53,54]. This, together with the previously mentioned diminished number of lymphocytes reported for the elderly, reflects the preference of

**TABLE 1**
**Age-Related Changes in Neutrophil Immunity**

| | References | (Possible) Reason |
|---|---|---|
| **Unchanged** | | |
| Spontaneous apoptotic death | 121$^{S}$ | |
| GM-CSF–mediated granulopoiesis | 56$^{S}$ | |
| Receptor expression (fMLPR, TREM-1, TLR2, TLR4) | 58$^{S}$, 82$^{S}$ | |
| **Increase** | | |
| Telomere shortening | 122$^{N}$ | ? |
| Membrane fluidity | 82$^{S}$ | ↓ Cholesterol, ↑ unsaturated phospholipids |
| Proinflammatory status | 105$^{S}$, 123$^{S}$ | ? |
| Basal $Ca^{2+}$ level | 82$^{2}$ | Increased adhesion |
| Adhesion after fMLP/PMA | 63$^{S}$ | Increased CD11b, CD15 |
| **Decrease** | | |
| Sensitivity to G-CSF, IFN$\gamma$ | 124$^{A}$, 125$^{A}$, | Disturbed signaling |
| L-selectin | 105$^{S}$ | Disturbed signaling? |
| Rescue from apoptosis towards (LPS, G-CSF, GM-CSF, IL-6, steroids, IL-2) | 55$^{S}$, 56$^{S}$, 57$^{S}$, 121$^{S}$ | ↓ Mcl2/Bax increase; ↓ caspase-3 inhibition; impaired antioxidant shield |
| Chemotaxis to fMLP, GM-CSF *in vitro* | 57$^{S}$, 58$^{S}$ | Disturbed signaling; ↓ actin polymerization |
| Impaired oxidant shield | 59$^{S}$ | ? |
| Phosphorylation in signaling pathways | 44$^{S}$, 56$^{S}$, 57$^{S}$, 82$^{S}$ | ? |
| $Ca^{2+}$ mobilization; second messenger generation ($IP_3$, DAG, PI, PIP, $PIP_2$, cAMP) | 55$^{S}$, 82$^{S}$ | ? |
| Actin polymerization after stimulation | 82$^{S}$, 126$^{S}$ | ? |
| TLR4/TREM-1 signaling/translocation of receptors into lipid rafts (TLR2/4, TREM-1, SHP-1) | 56$^{S}$, 57$^{S}$, 62$^{S}$ | Membrane fluidity; ↓ actin polymerization |
| fMLP signaling | 50$^{S}$ | ↓ Second messenger; ↓ phosphorylation of signaling molecules |
| GM-CSF signaling | 50$^{S}$ | ↓ Phosphorylation of signaling molecules; ↓ SHP-1 emigration from rafts |
| **Controversially** | | |
| Total number in circulation | → 51$^{S}$, 54$^{N}$, 74$^{A}$; ↓ 105$^{S}$; ↑ 126$^{S}$ | *In vivo* vs. *in vitro;* different measuring techniques; high rate of apoptotic cells at site of infection |
| $O_2^-/H_2O_2$ | → 84$^{S}$, 82$^{S}$; ↓ 64$^{S}$, 83$^{S}$, 90$^{S}$, 59$^{S}$ | Differences in duration time; *in vivo* vs. *in vitro;* time point after infection |
| Phagocytosis | ↓ 63$^{S}$, 69$^{N}$, 83$^{N}$, 73$^{S}$, 80$^{S}$; ↔ 78$^{S}$ | Differences in time point after infection starts; particles vs. microorganisms |
| Chemotaxis/tissue infiltration; *in vivo* vs. *in vitro* | 63$^{S}$, 76$^{A}$ | Differences in measuring techniques (output), time points after start of infection, species, high amount of apoptotic cells at sight of infection |
| **Unknown** | | |
| Cytokine expression; MHC expression | | |

↓ = decreased; ↑ = increased; ↔ = unchanged; ? = no published explanation.

Indices: $^{S}$ = according to SENIEUR protocol; $^{N}$ = nonselected elderly; $^{A}$ = animal model.

HSC of aged donors to differentiate into myeloid precursors[10]. The constant PMN number throughout aging suggests that functional changes lead to the reported failure of the innate immune system in the elderly.

There are no reports in the literature showing pathogenic delay in apoptosis or changes in CD95 expression on neutrophils of aged subjects. In contrast, several studies report that rescue from apoptosis is disturbed. Neither G-CSF, GM-CSF, IL-2, IL-6, TNF-$\alpha$, nor steroids or LPS can extend the life span of PMN from aged subjects[55,56,57]. One possible explanation is the changes in the ratio of anti- and proapoptotic members of the B-cell leukemia(Bcl)–2 family, such as Bax, Bad, myeloid cell leukemia (Mcl)-1, or BclXL. Whereas the Mcl-1/Bax ratio in neutrophils from young donors increases after GM-CSF stimulation, it remains unchanged in PMN from aged volunteers[40,58]. The Jak/Stat and MEK/ERK pathways, which regulate the expression of the Bcl-2 family members, are described to be defective in PMN from elderly subjects[40,50]. These findings and the disturbance of caspase-3 inhibition indicate that changes in signaling pathways may be a reason for the defective rescue from apoptosis.

Finally, the impaired antioxidant shield described for PMN of aged donors leads to an augmented cell oxidative load, which contributes to the shorter life span and early apoptosis[59]. Although superoxide dismutase (SOD) added exogenously to cell cultures was able to prolong neutrophil survival in both young and aged individuals, high amounts of the enzyme showed a still-increasing effect only in cell cultures of young donors[59].

Shortened life span and early apoptosis not only reduced the period of PMN activity, but may also lead to a high number of apoptotic PMN at the site of infection, which cannot be fully cleared by macrophages. This accumulation of PMN could progress into secondary necrosis (Fig. 1), finally ending in persistent chronic infections or frailty[38,60,61,62], conditions associated with aging.

## Chemotaxis

In case of an infection, PMN from peripheral blood are attracted by a gradient of chemotactic molecules, such as complement C5a, IL-8, or bacterial peptides like fMLP. To enter the infected tissue, PMN roll along and then firmly attach to the endothelial cells as depicted in Fig. 1. This is mediated through sequential interaction of CD15 and the integrins (CD11a/CD18 and CD11b/CD18) of PMN with P- and L-selectin, as well as ICAM-1, on the endothelial cells. There are numerous *in vitro* reports, indeed, showing a decrease in chemotaxis of PMN from elderly donors in comparison to chemotaxis of PMN from young donors, especially as response to fMLP and GM-CSF[38,58,63,64,65,66,67,68, 69,70,71,72,73 and Fig. 1]. One reason could be the lower actin polymerization of PMN derived from elderly donors after fMLP stimulation[71].

Changes in signaling pathways could also be involved. Consequently, a decreased infiltration of infected tissue by PMN should be anticipated. Surprisingly, *in vivo* studies in mice did not confirm this assumption. Several groups showed a normal infiltration in aged compared to young mice in a small excisional wound model generating a local immune response[74,75]. Other groups that elicited a systemic response through intraperitoneal injection of LPS[47] or a 15% total body surface area burn injury[76] even observed an increased organ infiltration by PMN in aged mice compared to that seen in young mice.

The conditions for studies with cells in suspension are quite different from the conditions for *in vivo* studies, since *in vivo* neutrophils, for instance, attach to endothelial cells or extracellular matrix in order to exert their function. The local accumulation of apoptotic PMN in infected tissue of the elderly could also account for the changes in migration detected *in vivo*. This, along with different measurement techniques, locations, and time points after initiation of infection, could explain the marked differences concerning *in vivo* and *in vitro* results. Furthermore, *in vivo* studies in humans usually occur at time points when infection is already advanced. Kovacs et al.[43] compared age-related changes in PMN of mice and humans and found a large array of discrepancies. They concluded that mice might not be the best model to study neutrophil immunosenescence[43].

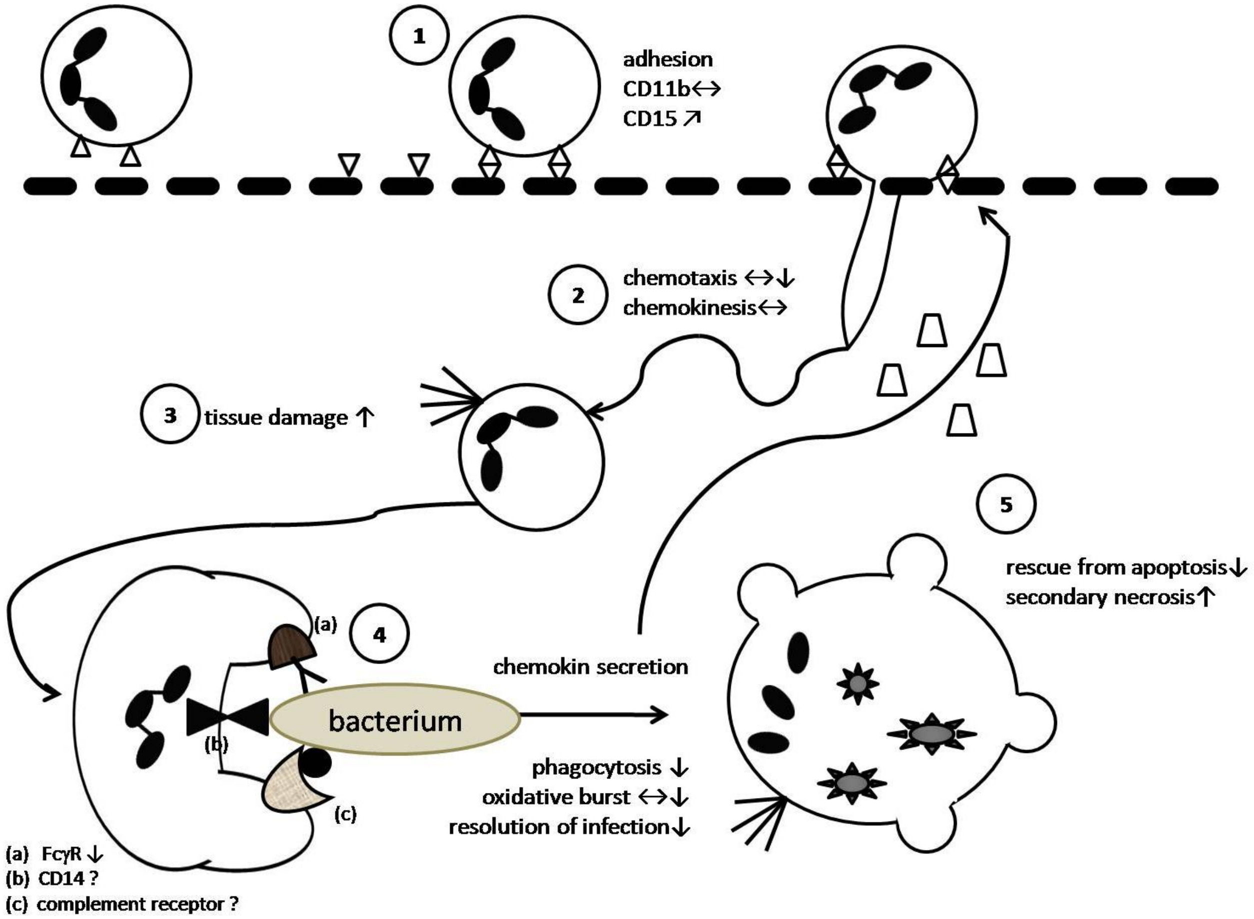

**FIGURE 1.** Main functional changes of PMN of the elderly. (1) Adhesion of PMN to endothelium and initial extravasation into tissues is constant or even slightly increased, probably due to slightly increased expression of CD11b and CD15. (2) Chemotaxis seems to be compromised by aging, while chemokinesis remains constant[73]. This reduces the efficiency of migration to the site of infection. (3) During migration through the tissue, PMN secrete proteases such as elastase from their granules. The reduction of chemotaxis would cause increased exposure to those proteases, consequently damaging healthy tissue. (4) Phagocytosis of microbes and subsequent killing by oxidative burst are probably reduced with age. This is partly due to decreased expression of Fcγ receptors, disturbed response to priming agents, and changes in underlying signaling pathways. The high rate of apoptotic PMN often cannot be cleared by macrophages, delaying the resolution of infection. (5) The impaired rescue from apoptosis described for PMN of aged donors leads to an increase of secondary necrosis and the leakage of intracellular proteins to the extracellular space and finally to inflammation. Each of those changes contributes to the reduced ability of the elderly to clear bacterial infections and to resolve inflammation. (Modified and adapted from Butcher et al.[79].)

More detailed research is necessary in this field to uncover the true PMN activities in the elderly. Unfortunately, so far, there are no analyses of the *in vivo* situation in humans that could answer the question whether discrepancies are due to differences between species. The unchanged chemotaxis shown in centenarians[63] suggests that the loss of this function could indeed be partly responsible for the failure of a proper immune reaction in the elderly.

## Adhesion

Adhesion, as an important step during migration, is reported to be unchanged or slightly increased. Several studies demonstrated that PMN from young and aged donors, stimulated with calcium ionophores, phorbol myristate acetate (PMA), zymosan, or fMLP, show the same ability to adhere to endothelium, gelatin, plastic, or nylon[69,77,78]. The expression of CD11a and CD11c by PMN, necessary for their extravasation[79], as well as the expressions of CD11a/CD18, CD11b/CD18, and CD14, are not altered or even slightly increased. The slightly increased expression of CD11b and CD15 could result in the enhanced adhesion of PMN from aged subjects to endothelial cell monolayers after stimulation with fMLP or PMA[63,64,80]. Consistently, Egger et al. found a general correlation of enhanced adhesion and impaired chemotaxis with higher susceptibility to infections[81].

Another consequence of the increased adhesion noticed in the elderly is a low, but constitutively increased, $Ca^{2+}$ level in the cytosol of unstimulated PMN from elderly donors[55,73]. fMLP usually leads to a transient increase of $Ca^{2+}$, but in the elderly, the mobilization of $Ca^{2+}$ from the endoplasmic reticulum into the cytosol or vice versa is reduced[82]. This results in a delayed maximal response to fMLP stimulation in comparison to PMN of young donors.

## Phagocytosis and Oxidative Burst

Whether there are age-related decreases in the phagocytic ability of neutrophils is still controversially discussed. Some studies show that phagocytosis is unimpaired in the elderly[78], others report a decreased phagocytosis of opsonized bacteria by neutrophils during aging[63,69,73,79,80,83,85]. Alonso-Fernandez et al.[63] additionally showed that the percentage of PMN that are able to phagocytose more than one particle (phagocytosis efficiency) also decreases[63]. Reasons for the decreased phagocytotic ability could be the changes in actin polymerization[82] or the significant age-related reduction of the Fcγ receptor (CD16), which is necessary for phagocytosis[79, Fig. 1].

In contrast, there are variable findings concerning changes in oxidative burst of PMN derived from aged individuals (Fig. 1). Some *in vitro* studies with PMN from aged donors describe a constant $O_2^-$ and peroxide production after stimulation with fMLP or gram-negative *Escherichia coli*[52,79,84]. However, more recent studies report that neither GM-CSF, LPS, TREM-1, and fMLP, nor the direct injection of gram-positive bacteria like *Staphylococcus aureus*, could fully activate intracellular killing[38,59,85,86,87,88,89,90,91,92]. Interestingly, after stimulation with fMLP or GM-CSF for 24 h, a reduced superoxide anion production is reported for PMN from aged donors, whereas it is even increased after stimulation for 48 h[58]. PMN that survived 48 h of fMLP stimulation were isolated and could be stimulated again. This experiment suggests the existence of a neutrophil subpopulation of “superPMN” in aged donors[58].

Whereas PMN from young donors seem to efficiently shut down the response to stimulation, PMN from aged donors seem to react more heterogeneously concerning intensity and duration of response[93]. These data suggest that the different outcomes of the studies are partly due to differences in timing of the analyses. Since the oxidative burst is a complex reaction and can be detected by the measurement of various parameters, the difference of results can also be due to different measuring techniques.

The production of antioxidants such as glutathione is also decreased in the elderly[63,94], as well as the ability to kill fungi like *Candida albicans*[86]. Both changes in phagocytosis and oxidative burst are possibly associated with alteration in $Ca^{2+}$ mobilization, membrane fluidity, and composition, as well as modifications in signaling pathways, which will be discussed later. Because phagocytosis and burst reactions are linked, and the disturbance of each of them or both together correlates with age-related disease, as well as with decreased antimicrobial capacity, detailed insights into their cross-talk are required.

PMN are able to synthesize several immunoregulatory proteins in addition to their known traditional role as professional phagocytes. Whereas our own results showed the synthesis of mostly anti-inflammatory or chemotactic molecules such as IL-8, IL-1 receptor antagonist, macrophage inflammatory protein (MIP)-1α, MIP-1β, sTNF-R, and growth-related oncogene (GRO)-α[39,40,95,96], others also detected IP-10, IL-17, IL-12[97,98,99], and proinflammatory IL-1 and TNFα [100,101]. Because low percentages of contaminating monocytes can yield false-positive data, especially in mRNA analyses, results have to be regarded critically[95,102]. However, since there are no reports showing age-dependent changes in cytokine production in PMN and since their capacity to produce cytokines is still discussed controversially, this is a topic to be investigated in the near future.

The increased proinflammatory cytokine production described in myeloid DC and monocytes of aged donors[19,37,103], along with the overall low-grade inflammatory condition in the elderly, suggests that cytokine production of PMN could also change during aging. Our own data showed an increase in the expression of proinflammatory cytokines together with changes in chromatin of the IL-1β gene into a transcriptional active state in at least monocytes from aged men, supporting possible changes in cytokine production due to aging[104].

Those alterations in cytokine production could also contribute to the low-grade inflammatory condition reported for resting neutrophils of aged volunteers, which is additionally affirmed by CD62L shedding and constitutive ROS production[77,105]. Furthermore, the existence of a basically proinflammatory condition is underlined by permanent phosphorylation of signal transduction molecules[44,106], as well as by the alteration of the distribution of signaling molecules in lipid rafts (LR) and a constantly increased $Ca^{2+}$ level in resting PMN from aged donors[50,58,71].

## PMN as Antigen-Presenting Cells

Unfortunately, there are no analyses of PMN from aged subjects concerning the expression of MHC-I, MHC-II, or CD80 molecules, even if those link neutrophils to the adaptive immune system[107,108,109,110,111]. Another surface molecule whose expression is altered through aging is ICAM-3 (CD50)[105]. De Martinis et al. describe a down-regulation of the density of CD50 on the surface of granulocytes of aged subjects at a "per cell" level[105]. Interestingly, the overall number of CD50-positive cells was increased in aged volunteers. Changes in the ability of PMN to process and present foreign antigens, and the accessory cell function of PMN of aged donors, still need to be investigated.

## Signaling Pathways

Functional changes detected in PMN from aged donors can be ascribed to alterations in signaling pathways and their receptors, as well as receptor distribution and alterations of membrane fluidity. As summarized in Fig. 2 and extensively reviewed elsewhere, signaling pathways of fMLP-R, GM-CSF-R, TREM-1, and TLR4 are all changed in PMN of aged donors[39,57]. Interestingly, the expression of these four receptors remains constant throughout aging, which is in contrast to age-related changes in TLR expression reported in monocytes[22], underlining that aging effects are different in various cell types.

Changes in fMLP-mediated signal transduction are mostly due to the nonadequate synthesis of the second messenger's diacylglycerol (DAG) and inositol triphosphate ($IP_3$)[82], as well as $Ca^{2+}$ mobilization[50]. The changes in second messenger generation become apparent in the decrease of chemotaxis and superoxide generation reported in PMN from aged donors.

The production of phosphatidic acid (PA), which activates NADPH oxidase, is intact in PMN of aged subjects. Superoxide generation was corrected if the intervening signal transduction steps were bypassed by administration of PMA. This together with the intact PA synthesis underlines the assumption that fMLP-mediated disruption of ROS production occurs early in the signaling cascade. The lower phosphorylation of p38MAPK and ERK1/2 of PMN from aged subjects are also reported alterations in fMLP-induced signaling[60, Fig. 2].

The failure of TLR4-induced signaling has a completely different background. TLR4 has to be recruited to LR in order to execute its function[52]. Its LPS-mediated translocation is interrupted in PMN from old donors. Unfortunately, no direct connections between failures of the TLR-mediated signaling and disrupted functions of PMN from aged individuals have been shown. Surprisingly, LPS-mediated chemotaxis seems to be intact. However, previously mentioned differences in results concerning chemotaxis in the elderly make clear that those connections should be further discussed.

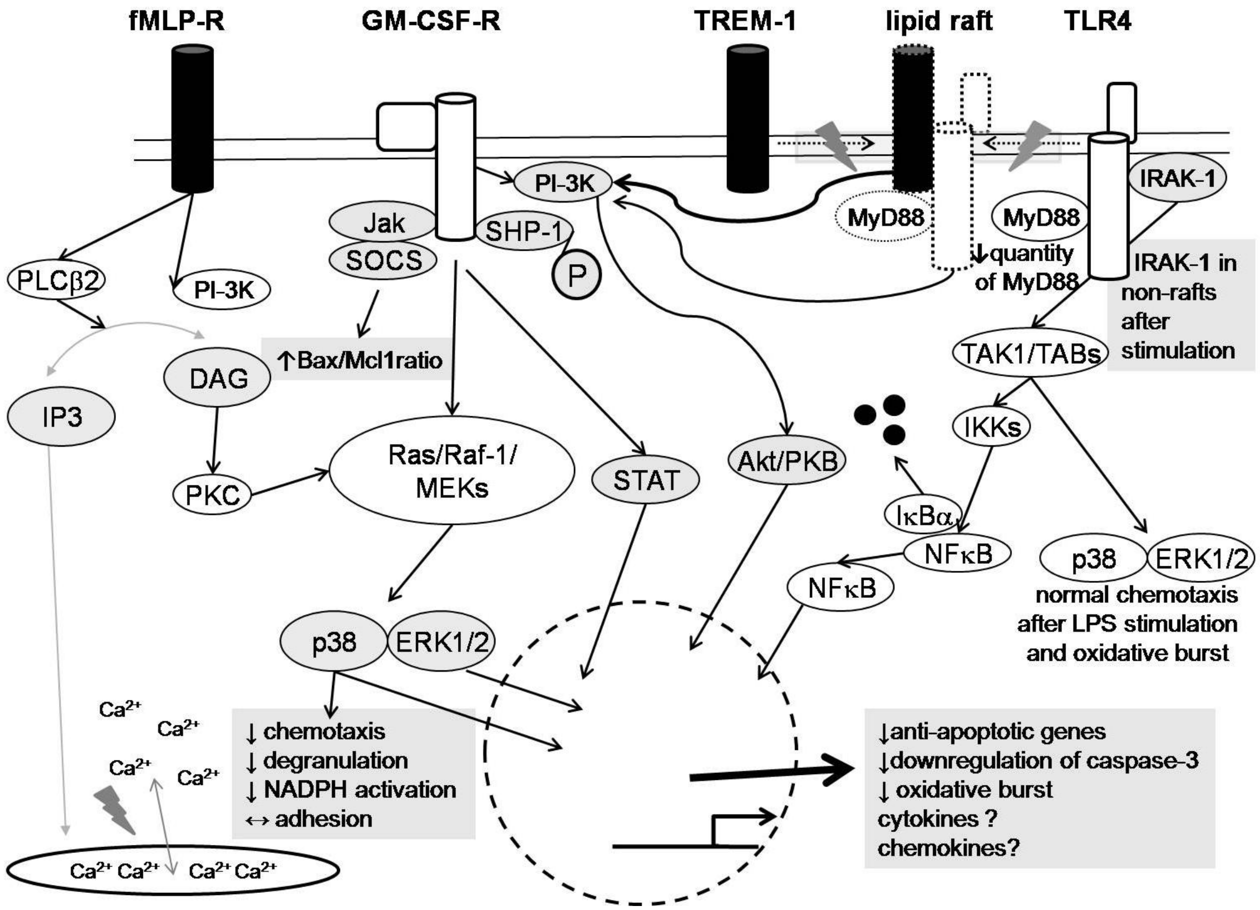

**FIGURE 2.** Signaling in neutrophils of the elderly. Age-related impairment in intracellular signaling after binding of the appropriate ligands to their receptors explains the functional changes in PMN of aged subjects. Molecules with gray background display defects in the signaling cascade associated with age. Translocation of TREM-1 and TLR4 into LR is indicated by dashed lines. Interestingly, signaling induced by ligands of fMLP-R, GM-CSF-R, and TREM-1 is mostly disturbed, while the majority of TLR4-mediated signaling remains intact throughout aging. (Modified and adapted from Uciechowski and Rink[39].) PLC, phospholipase C; DAG, diacylglycerol; IP3, inositol triphosphate; MEK, MAPK (mitogen-activated protein kinase)/ERK kinase; ERK, extracellular signal-regulated kinase; PKC, protein kinase C; PI-3K, phosphoinositide-3 kinase; TREM-1, triggering receptor expressed on myeloid cells.

The knowledge of the newly discovered TREM-1 is limited. For its appropriate function, it has to be translocated into LR, too, where it colocalizes with TLR4 or the NACHT-LRR (NLR) pattern recognition receptor[46,107]. In PMN from the elderly, TREM-1 remains in the nonraft fraction of the plasma membrane. This is a possible explanation for the impaired TREM-1–induced functions reported for PMN of aged volunteers. The disturbance of the interaction with the NLR receptor, which recognizes intracellular pathogens, abolishes the synergistic induction of cytokine production. Because the interaction with TLR4 is also affected, the defense against extracellular pathogens is weakened through abolishing ROS production and the missing extension of life. Reasons for the defects in translocation of TLR4 and TREM-1 could be the reduction in actin polymerization, but also the increased fluidity of the plasma membrane, which also effects the integrity of lipid rafts[52].

All three pathways activated through GM-CSF-R ligation are disturbed in the elderly[42,52, Fig. 2]. These disruptions are primarily due to the failure of phosphorylation of signaling molecules throughout the signaling cascade. The interruption of GM-CSF signaling can also be a consequence of decreased actions of signal tyrosine phosphatase-1 (SHP-1)[56]. In contrast to the inhibited recruitment of TLR4 and TREM-1, the action of SHP-1 is blocked by disturbance of its emigration from LR[72,56]. The primary consequence of disturbed GM-CSF–mediated signaling in PMN of aged donors is the inability to be rescued from apoptosis. This is partly because of changes in caspase-3 cleavage, but also on account of the deregulation of the ratio of pro- and antiapoptotic members of the Bcl-2 family. Age-related changes in respiratory burst and degranulation of PMN can also be ascribed to defective GM-CSF signaling pathways. Because GM-CSF can also prime PMN to a second stimulation just like fMLP, the disruptions of its signaling are even more detrimental for normal PMN functioning.

Alvarez et al.[108] investigated the influence of the changed fluidity of the fMLP-induced ROS generation and found correlation between increased membrane fluidity and decreased ROS production. This is in contrast to the finding that PMA, which activates ROS generation directly via PKC, functions independently of membrane fluidity[109]. It suggests that the defects detected in the elderly are proximal to the receptors. Unfortunately, the association of GM-CSF signaling and membrane composition has not been studied yet. The defects in signal transduction pathways, which result in an increased susceptibility to apoptosis, may be important contributors to aberrant inflammatory responses during senescence.

## PMN, Aging, and Disease: Causes and Consequences

Whereas the association between age-related changes in neutrophils and diseases is well described[62], the mechanisms that result in age-related changes of PMN are mainly speculative. There are six basic theories that try to give reasons for age-related declines in cell functions, thoroughly discussed by Wilson et al.[112], namely: ‘‘wear and tear’’ theory, “Hayflick limit (or ‘ageing clock’)” theory, “genetic control” theory, “error catastrophe” theory, and “free-radical theory”[112]. The latter states that age-related changes in the immune system are due to life-long exposure to oxidative stress[63], causing accumulation of several forms of DNA damage and mutations[112]. This can result in decreased expression of surface receptors or transcription factors, causing defects in transmigration, pathogen recognition and phagocytosis, or signaling pathways, respectively. Centenarians show well-preserved antioxidant ability in contrast to normal elderly subjects whose low superoxide production suggests that oxidative stress of PMN, but also other immune cells, could form the basis for functional disturbances in the elderly[63].

Epigenetic modifications can also be attributed to molecular changes that result in cellular diversity and function. The association of epigenetic changes with Alzheimer’s disease[113] and T-cell dysfunction[114] has already been reported. During aging, the overall content of 5-methyl-cytosines declines, while several promoter DNA regions are hypermethylated in a tissue-specific way[114,115]. This indicates that epigenetic alterations could also account for functional changes in PMN of aged donors.

Inadequate nutritional supply has been shown to contribute to an impaired immune response[116], causing impaired phagocytosis, parasite killing, oxidative burst, and thymus involution[116], resembling age-related disturbances. Zinc deficiency, as an example, increases susceptibility to *Salmonella enteritidis*, *Mycobacterium tuberculosis*, or *Listeria monocytogenes*[117]. One major mechanism by which zinc affects immunity is its influence on the immune cell signal transduction. Zinc is required for the activities of the kinases Lck and phosphokinase C in T cells[118]. In monocytes, zinc regulates signaling cascades via influencing cyclic nucleotide phosphodiesterases and MAPK phosphatases[118]. A recent study identified that zinc also takes part in NF-κB signaling in peripheral blood mononuclear cells (PBMC)[119]. Hence, changes in signaling due to zinc deficiency remarkably parallel those seen during aging. So far, there are no data showing an effect of zinc on signaling in PMN, but the results for T cells and monocytes indicate that zinc deficiency in the elderly could cause changes in PMN via influencing signaling.

Finally, nutrients are important cofactors or substrates for enzymes, such as acetyl- or methyltransferases, necessary for epigenetic modifications and regulation of gene expression[115]. Transcription is also regulated by amino acids because there are amino acid response elements in promoter regions of certain transcription factors[120].

The epithelial lining of the gut of elderly subjects is frequently compromised, enabling a great influx of foreign antigens into the portal circulation from the intestine to the liver. There, the locally increased antigen load contributes to a chronic inflammatory condition, which in turn again causes changes and defects in PMN[75].

However, since PMN are at the beginning of a chain of recruitment, differentiation, and activation of both innate and adaptive immune cells, their defects accelerate the progression of the disturbance of the immune response. Therefore, several age-related diseases that are obviously caused by disturbances of other immune cells may be based on deregulation of PMN.

Even if recent studies highlight some of the molecular mechanisms underlying the functional changes in PMN of aged donors, there are still questions to be answered. There are, to our knowledge, no published explanations for the decrease in phosphorylation of signaling molecules, actin polymerization, or second messenger generation. Additionally, it has to be clarified by which mechanisms membrane composition is changed during aging. Knockdown or inhibitor experiments could be useful approaches to detect responsible molecules. Alternatively, the study of persons with genetic signaling defects would be helpful to get insight into the *in vivo* situation.

## CONCLUSION

Several aspects of PMN responses are affected by normal human aging, including traditional PMN functions such as phagocytosis and oxidative burst. These changes result in the reduced ability to eliminate bacteria and fungi, but also inhibit the interaction with and influence on the adaptive immune system. The mechanisms underlying these changes are now beginning to be characterized and include primarily changes in receptor assembling and signaling pathways. The possibility of restoring functions in PMN of aged donors via bypassing distinct signaling pathways not only elucidates the association of a defect to a certain signaling pathway, it also opens the way for drug development in order to prevent or cure age-related defects of human PMN and thereby preserve human health. The production and secretion of immunomodulating molecules has not been analyzed in an age-dependent context, either. Additionally, analyses of MHC expression in the elderly are missing. Even if experiments in mice could provide useful insights into the situation of PMN of the elderly *in vivo*, the transfer of those results to the situation in humans remains debatable.
